# Yellow Fever

**Published:** 1898-08

**Authors:** 


YELLOW FEVER. Clinical Notes by Just Touartne, M.D. (Paris). Translated from the French manuscript by Charles Chassaignac, M.D., New Orleans. New Orleans Medical and Surgical Journal, 1898.

This is a most thorough clinical study of yellow fever, and to our thinking, the most accurate description of the disease, in all its phases, it has been our pleasure to read. The author, in a practice of thirty years at New Orleans, has had abundant opportunity to study the disease, and he has used his opportunities.

One point which he brings out in the symptomatology should be emphasized, viz.: that yellow fever is a disease of *one paroxysm*, and that the third stage, or second exacerbation, described in text-books is a myth, occurring only as a result of some complication. In the recent work of Izett Anderson, "Yellow Fever in the West Indies," the same position is taken, and this is in accord with our observation.

Dr. Touartne's suggestions as to prevention and treatment are thoroughly practical.

He says "the sovereign remedy in yellow fever will be the antitoxin whose discovery is announced by Sanarelli." Which suggests, why not use the antitoxin prepared by nature in the blood of persons recovered from the disease, as proposed by Sternberg?

As the subject of yellow fever is likely to interest us of the United States for years to come, it would be well for physicians likely to come in contact with it to study this work of Dr. Touartne's. C. C. S.

---

### AMERICAN MEDICAL ASSOCIATION.

At the recent meeting of this Association the following was unanimously adopted:

Whereas, the American Medical Association did, at Detroit in 1892, unanimously resolve to demand of all the medical colleges of the United States the adoption and observance of a standard of requirements of all candidates for the degree of doctors of medicine which should in no manner fall below the minimum standard of the Association of American Medical Colleges; and

Whereas, this demand was sent officially by the Permanent Secretary to the deans of every medical college in the United States and to every medical journal in the United States, now therefore the American Medical Association gives notice that hereafter no professor or other teacher in, nor any graduate of any medical college in the United States, which shall after January 1, 1899, confer the degree of doctor of medicine or receive such degree on any conditions below the published standard of the Association of American Medical Colleges, be allowed to register as either delegate or permanent members of this Association.

Resolved, that the Permanent Secretary shall within thirty days after this meeting send a certified copy of these resolutions to the dean of each medical college in the United States and to each medical journal in the United States.

